# Conceptualisation of job-related wellbeing, stress and burnout among healthcare workers in rural Ethiopia: a qualitative study

**DOI:** 10.1186/s12913-017-2370-5

**Published:** 2017-06-19

**Authors:** Medhin Selamu, Graham Thornicroft, Abebaw Fekadu, Charlotte Hanlon

**Affiliations:** 1 Department of Psychiatry, Addis Ababa University, College of Health Sciences, School of Medicine, PO Box 9086, Addis Ababa, Ethiopia; 2 Centre for Global Mental Health and Centre for Implementation Science Health Services and Population Research Department, King’s College London, Institute of Psychiatry, Psychology and Neuroscience, London, UK; 3 Department of Psychological Medicine, King’s College London, Institute of Psychiatry, Psychology and Neuroscience, Centre for Affective Disorders, London, UK; 4 Health Services and Population Research Department, King’s College London, Institute of Psychiatry, Psychology and Neuroscience, Centre for Global Mental Health, London, UK

**Keywords:** Stress, Stressor, Job related stress, Burnout, Wellbeing, Healthcare workers, Primary care, Ethiopia

## Abstract

**Background:**

Wellbeing of healthcare workers is important for the effective functioning of health systems. The aim of this study was to explore the conceptualisations of wellbeing, stress and burnout among healthcare workers in primary healthcare settings in rural Ethiopia in order to inform the development of contextually appropriate interventions.

**Methods:**

A qualitative study was conducted in a rural zone of southern Ethiopia. A total of 52 frontline primary healthcare workers participated in in-depth interviews (*n* = 18) or Focus Group Discussions (FGDs) (4 groups, total *n* = 34). There were 35 facility based healthcare professionals and 17 community-based health workers. Data were analysed using thematic analysis.

**Results:**

Most participants conceptualised wellbeing as absence of stress rather than as a positive state. Many threats to wellbeing were identified. For facility-based workers, the main stressors were inadequate supplies leading to fears of acquiring infection and concerns about performance evaluation. For community health workers, the main stressor was role ambiguity. Workload and economic self-sufficiency were a concern for both groups. Burnout and its symptoms were recognised and reported by most as a problem of other healthcare workers. Derogatory and stigmatising terms, such as “chronics”, were used to refer to those who had served for many years and who appeared to have become drained of all compassion. Most participants viewed burnout as inevitable if they continued to work in their current workplace without career progression. Structural and environmental aspects of work emerged as potential targets to improve wellbeing, combined with tackling stigmatising attitudes towards mental health problems. An unmet need for intervention for healthcare workers who develop burnout or emotional difficulties was identified.

**Conclusion:**

Ethiopian primary healthcare workers commonly face job-related stress and experience features of burnout, which may contribute to the high turnover of staff and dissatisfaction of both patients and providers. Recent initiatives to integrate mental healthcare into primary care provide an opportunity to promote the wellbeing of healthcare workers and intervene to address burnout and emotional problems by creating a better understanding of mental health.

## Background

The wellbeing of healthcare workers (HCWs) is important for the effective functioning of health systems. High levels of job-related stress can affect the wellbeing of HCWs adversely, leading to mental health problems and experience of burnout [1].Burnout is a global occupational hazard among HCWs and other human service professionals [2, 3]. Burnout is conceptualised as comprising emotional exhaustion, distancing oneself from patients and reduced feelings of personal accomplishment [4]. In studies from high income countries, the prevalence of burnout among HCWs ranges from 12.6% [5] to 29.9% [6]. In a large survey of mental health professionals in the United Kingdom, emotional exhaustion was reported to affect 20.1% [7]. Although there have only been a few reports from low and middle income countries (LMICs), the prevalence of burnout has been found to be much higher; for example, affecting 68.0% of nurses in Tunisia [3]. Mental health problems also appear to be common. In a study from a tertiary level health facility in Ethiopia, 29.9% of HCWs reported mental distress [8]. These indicators of compromised wellbeing may be related to high levels of job-related stress in under-resourced settings. Job related stress was reported by 52.7% of primary care medical officers in a study from Pakistan [9].

Worldwide there is a shortage of HCWs [10] which is particularly acute in sub-Saharan Africa (SSA) [11, 12]. In the 2007 World Health Report, it was estimated that African healthcare systems provide care to address the world’s most substantial disease burden (24%) with only 3% of the global HCWs and scant financial resources [13]. In LMICs, HCWs are exposed to poor wages, low socioeconomic status [2, 14], high workload, role ambiguity, challenging working conditions [3, 14], limited support in handling disturbed patients [15] and poor management [16]. These factors are also shared by studies in high-income countries [17, 18]. Threats to the wellbeing of HCWs in SSA are, therefore, substantial.

Compromised HCW wellbeing has important implications for the health system. High staff turnover [19] and ‘brain drain’ is an ever-present challenge to African health sector human resources; both brain-drain to more wealthy countries and also in-country brain drain to the private or non-governmental sector [20]. This adds greatly to healthcare organisational costs due to the need for recruitment and training of new HCWs. Staff wellbeing may be an important factor contributing to staff turnover, but there has been little exploration of this relationship in sub-Saharan Africa to date [16]. Health service quality is also highly influenced by the skill and enthusiasm of staff, as well as their number and the availability of material resources [21]. Indeed HCW wellbeing and patient safety are strongly associated [22]. There is evidence that the poor quality of health services in sub-Saharan Africa is related, at least in part, to the job satisfaction and level of motivation of HCWs, both of which are associated with wellbeing [23].

Ethiopia is moving towards expansion of healthcare coverage for all, but is still a disadvantaged country on most health parameters, including life expectancy, maternal mortality and infant mortality [24]. There is a high level of unmet need for health services [25], which is exacerbated by the shortage and high turnover of staff [26, 27]. In the Ethiopian Health Sector Transformation Plan (HSTP), the need to develop ‘caring, respectful and compassionate’ HCWs is emphasised as a means to transform primary care [28]. HCW wellbeing is critical to achieving this goal, but the construct of wellbeing has not been investigated adequately in low-income countries like Ethiopia. Findings from high-income countries may not be generalisable due to differences in expected HCW roles, in particular the strong reliance on task-shifting to lower cadres of healthcare worker in LMICs, the level of disease burden and the weak health system infrastructure. Investigation of wellbeing of both community- and facility-based primary HCWs in rural Ethiopia is both timely and necessary.

The aim of this qualitative study was to explore conceptualisations of job-related stress, burnout and wellbeing amongst primary HCWs in Ethiopia, in order to inform the future development of an intervention to promote their wellbeing.

## Methods

### Study setting

The study was conducted in the Silte Zone of the Southern Nations, Nationalities and Peoples’ Regional state of Ethiopia (SNNPR). The Silte Zone has a total population of approximately 750,000 [29]. In keeping with most rural Ethiopian settings, the health service in the zone is provided by the government, with a focus on primary care. The zone has a total of 33 primary healthcare (PHC) facilities in the public sector, each serving 15 to 25,000 people. The study site was selected for the following reasons: facilities in the Zone are typical of rural PHC settings in Ethiopia, the research team has an established relationship with the district health office administration and the zone is in close proximity to Sodo district which is the site for the next phase of the study [30].

Almost all public PHC facilities are staffed by health officers and nurses, usually with no doctors [31]. Health officers are Bachelors level first degree holders, trained for three to 4 years. They are trained to be an intermediate between medical doctors and diploma nurses. There are also some degree level nurses who are trained for three to 4 years, but most frontline nurses are diploma level, with only 2 years of training. Health officers and both levels of nurses are expected to see patients and to manage all types of cases, with a focus on outpatient care, as well as to support the health extension workers (HEWs). HEWs are trained for 1 year after completion of high school (tenth grade). Their work is community-based and they are mostly engaged in prevention and health education work. In addition, they are expected to run satellite, community-based clinics called health posts, which provide family planning, vaccinations and other related preventive services.

The present study was nested in an international project, the Programme for Improving Mental health care (PRIME) involving five LMICs, including Ethiopia [32]. PRIME aims to develop evidence for the provision of mental health services in primary healthcare and is being implemented in the Sodo district of the Gurage zone, which neighbours the Silte zone [32, 33]. The PRIME service approach [30] is informed by the National Mental Health Strategy of Ethiopia [34] in which primary HCWs will receive brief training and periodic supervision to provide mental healthcare in a task-sharing model of care [35]. This study was conducted as part of PRIME because previous studies have indicated that HCW wellbeing is essential for delivery of quality mental health care [36], and to inform potential interventions that could be integrated into the PRIME scale-up to promote and improve HCW wellbeing [30].

### Study design and sample

A qualitative study was used because the concepts of wellbeing, job-related stress and burnout have not, to our knowledge, been explored previously in frontline PHC workers in LMICs, particularly community based workers, such as HEWs. In-depth interviews (IDIs) and focus group discussions (FGDs) were used. A semi-structured topic guide was employed for the interviews. The rationale for using both FGD and IDIs was to obtain a range of information and to triangulate the information from different data sources. We anticipated that some topics would be sensitive, for example, disclosing emotional problems or discussing difficulties in the relationship with senior managers, and best addressed in an IDI. On the other hand, FGDs allow for exploration of how concepts around wellbeing are talked about in front of others and the relative priorities given within the group, as well as the extent to which the concepts are shared by different types of HCWs.

The study participants were selected purposively in order to investigate perspectives by gender, place of work (facility-based or community-based), type of training and levels of experience. FGDs were organised in two groups, one for community based workers and one for facility-based workers. For each FGD, participants of similar level were grouped together to maximize homogeneity. The final sample size was decided by the point at which theoretical saturation was obtained [37]. The inclusion criteria were: (1) working in a PHC setting in the Silte zone, (2) being engaged in direct patient care or supervision and planning, and (3) having a minimum of 3 months work experience in the current work place or similar setting.

### Data collection procedures

Prior to data collection, permission to carry out the study was obtained from the Zonal Health Office. The Zonal Health Office provided a list of all HCWs working in their zonal health centres and health posts. Data was collected from May up to June 2014.

All IDIs were conducted by the first author (MS). MS also moderated all FGDs and was assisted by experienced note-takers. With the consent of the participants, all IDIs and FGDs were audio-recorded. The interviews and focus group discussions were conducted in Amharic, the official language of Ethiopia. The audio records were transcribed in Amharic and then translated into English for further analysis and write up. The translation was checked by one of the researchers to ensure that the concepts had been translated correctly. All the IDIs and FGDs were carried out either in health centres where the HCW was working or in a nearby health centre. Nurses and health officers were interviewed in their workplace, but HEWs were invited to come to a nearby health centre. During the IDIs and FGDs the Amharic terms “*kesera gar yeteyayaze metaket*” (job related emotional and physical exhaustion or job related lassitude) and “*weteret/chenket*” (tension or unable to relax/being anxious) were used to describe burnout and stress, respectively. Apart from the two FGDs with HEWs, people working in the same health centre were grouped together.

### Data analysis methods

Data analysis was carried out alongside ongoing data collection and an interim analysis informed adaptation of the topic guide and ongoing sampling. To analyse the data, thematic analysis [38] was used. We employed a rigorous and systematic approach [39]: *Step 1*: The English transcripts were read repeatedly by MS and last author (CH) to ensure familiarity with the data. *Step 2*: After independent coding by co-authors MS and CH, there was a discussion which led to development of categories and a coding plan. *Step 3*: MS and CH tested the codes for clarity and consistency by selecting a given text. *Step 4*: MS coded the rest of the text and identified emerging themes as the data were still being collected. *Step 5*: MS and CH reassessed coding consistency. *Step 6*: MS interpreted the coded data and described the themes and categories of the data. *Step 7*: MS summarised the report including the method followed in order to make the study replicable [40–42]. NVivo 9 software was used to support the analysis and management of the data [43]. To ensure the credibility of the analysis, member checking was done with three study participants [44, 45].

### Ethical considerations

Ethical approval was obtained from the Institutional Review Board of the College of Health Sciences, Addis Ababa University. All participants gave written informed consent. Participants who experienced any sort of emotional disturbance or mental health problem were informed about how they could obtain care.

## Results

### Participants

The sample comprised five BSc degree nurses, 23 diploma nurses, five health officers and 17 health extension workers (HEWs). There were 22 males and 30 females, with 17 working in health posts and 35 working in health centres. Two participants were health centre heads and the rest were frontline staff. The IDIs took a minimum of 35 and maximum of 90 min. The FGDs took a minimum of 60 and maximum of 120 min. The size of the FGDs ranged from 7 to 10 participants.

**Fig. 1:**
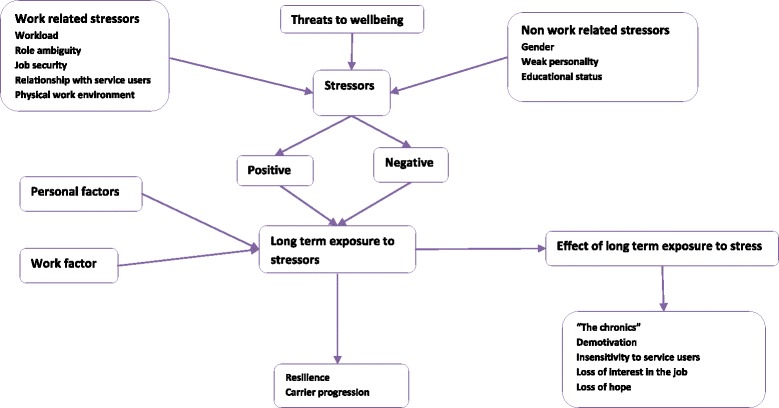
Primary Healthcare worker’s conceptualisation of Job related stress, burnout and wellbeing

The sociodemographic characteristics of the participants are presented in Table 1. Due to the consistency of information between IDIs and FGDs we have presented the results together, highlighting differences where present. The main results of this study are presented under the identified three themes. The conceptual model of this study is presented in Fig. 1. Additional quotes under the identified themes are included in the supplementary file.

**Table 1:** Socio demographic characteristics of study participants

	IDIIDI		FGDDFGDFGD	
	Male	Female	Male	Female
Number of participants involved	11	7	12	22
Age (years)				
< 25	1	3	4	13
25–34	8	4	7	9
35–44	1	-	-	-
> 44	1	-	1	-
Marital status				
Married	4	3	-	16
Single	7	4	-	6
Educational background				
Health officer	3	2	1	1
BSC nurse	3	1	1	-
Diploma nurse	4	3	10	6
HEW	-	2	-	15
Year of service				
< 1 year	1	2	2	1
1–3 years	4	1	1	7
> 3 years	6	4	9	14

### Theme one: Wellbeing and threats to wellbeing

#### Wellbeing

Wellbeing was more often explained as the absence of negative things in the HCW’s life rather than being described in relation to its positive attributes. For example, wellbeing was equated with workplace safety from infection and security from violent caregivers or patients. However, a few respondents had positive descriptions of well-being: being accepted and having a good relationship with the community, being healthy and economically self-sufficient. The descriptions of wellbeing lacked depth and contrasted to the rich explanations given about the threats to wellbeing.

#### Threats to wellbeing

Stressors related to the job were not considered to be uniformly negative. Most participants agreed that some degree and type of stress was acceptable and unavoidable in healthcare. Working night shifts and on public holidays, treating patients who are in pain and sharing distressing moments of patients and caregivers were some of the acceptable stressors. Some said that HCWs are expected to be strong to handle those stressors, but others spoke of the profession as being too demanding. A participant described his experience as follows:

*We are sacrificing our life to give life to the patients. We have much contact with patients who are facing diverse problems…we are listening to their problems and watching when they [patients] are in pain. Eventually this will pressure us and create some stress and make us unhappy…there are situations that demands our immediate action but we can’t… which is quite stressful* [FGD four, code 07]

All of the participants shared the view that exposure to stressors is acceptable for a certain period of time. However, across the interviews and FGDs, it was indicated that prolonged exposure to stressors might progress to an unacceptable or negative stressor and lead to emotional disturbance. Participants also spoke about personal life stressors, such as competing family responsibilities like child care, as well as being unable to fulfil family demands due to low income or having a physical illness. Most of the participants presented general life stressors and job-related stressors as overlapping and interconnected. For example the imbalance between the demands of the job and their income was presented as both a personal life and job-related stressor.

Emotional consequences of exposure to stressors (“being stressed”) were related by participants to the characteristics of the individual. Participants implied that HCWs who displayed any type of emotional response to stressors were somehow weak or showing ‘instability’. For example:

*If the person is thinking too much or if the person is worried about everything he can be stressed. …it can also be caused by [mental] instability* [IDI code 06]

Workload and lack of clarity about the boundaries of their role were mentioned as stressors. Facility based participants said that outreach work, which involves walking long distances, was a cause of exhaustion. Community based workers spoke of the lack of definition to their roles and workload as the major sources of stress. A participant shared her experience:

*We are responsible for most of the activities in the sub-district, be it health or agricultural issues. We are working under great pressure. When there is new fertilizer we are supposed to introduce it… when there is primary school registration we are the ones who will go house to house and register… I sometimes feel difficulty to see the boundary of my responsibilities* [IDI code 05]

Most participants described how the relationship with patients and co-workers, including superiors, could either be a stressor or protector. The majority of participants reported a positive and harmonious workplace relationship with co-workers that had helped them to handle stressors. Some indicated that they were part of a close-knit team, with supportive and encouraging relationships that gave them joy in their workplace. Perspectives on relationships with superiors were more mixed, with most giving the impression that they were indifferent. All facility based participants mentioned that their superiors or health centre leaders were distant because they are very busy, but most appeared reluctant to discuss the topic openly.

The relationship with the patients and caregivers was reported as difficult by most respondents, with only a few considering it to be good. Respondents reported being undervalued by the community, especially after the government’s recent introduction of HCWs evaluation by patients, community members and government officials. All reflected that this monitoring and evaluation system had made their relationship with the community challenging and contributed to feelings of insecurity. Some reported being discouraged or frustrated, particularly as the community evaluation carried punitive consequences:

*During a community forum, any community member can accuse you of anything… and you will be demoted to very remote place without getting a chance to explain ourselves, we are uncertain what will happen to us*. [FGD one, code 02]

In contrast, one participant spoke positively about the community evaluation system:

*When they [patients] face a delay or any sort of inconvenience or mistreatment they know how to complain about it....They will raise the issues in the community forum which is very helpful for the improvement of the health service. It is really helpful system; they can tell us what we cannot observe*. [IDI code 04]

The physical workplace conditions especially infrastructure such as tap water, phone signal, access to public transportation and availability of medical supplies were mentioned as stressors by many participants. Many participants said that shortage of medical supplies was one of the worst stressors, mainly due to fear of acquiring infection. One participant described the situation with respect to shortage of gloves as follows:

*If you want to work properly and protect yourself…you are supposed to persuade the family to buy gloves…but sometimes we expose ourselves in order to save their money.... our work environment is not comfortable, it is not safe, it is stressful.* [IDI code 01]

However, participants who were involved in management and leadership reported that there were adequate medical supplies.

### Theme two: “the chronics”

According to participants, long-term exposure to stressors had a critical impact on the HCW. They indicated that stagnating in one place without career progression can lead to long-term exposure to stressors. This was seen as a pathway to becoming a “chronic” member of staff, which was a label given to healthcare workers who have served for many years in the same post. This term carried a negative connotation for the bearer of the label. Although most respondents identified the effects of long-term exposure to stressors, most spoke of it as a problem of other HCWs. One participant shared her observation of a ‘chronic’ staff member as follows:

*There was a HCW ‘who adapted to other person’s pain’ … The incident was like this… There was a female patient who was being catheterized [urinary catheter]; the way he inserted the catheter was very inhumane. I was feeling the pain; my eyes were filled with tears … but this person was a senior professional. At that time he was treating the lady as something without sense… he was like a carpenter who is hammering a wood. The patient was shouting but he was not able to notice that… he was just doing his job without being disturbed.* [IDI code 02]

Another participant described this group as follows:

*These are very bored professionals…those who are paid less, especially the ‘chronic ones’ are very bored and exhausted. We [BSc level professionals] earn better than them. Most of them are supposed to support their families, the financial burden has dissatisfied them and they work carelessly* [FGD four, code 06]

All except two respondents said that they had encountered the consequences of long-term exposure to stressors in other HCWs. The terms “burnout” and “burnout syndrome” were not familiar to most of the participants; however, they described in detail how HCWs can become demotivated, insensitive to patients, lose interest in their job, feel exhausted when treating patients and lose hope. Most said that they have encountered someone in their workplace experiencing those problems. A participant described it as follows:

*The consequence… is loss of hope. I have never experienced it but I can tell you about my friends. I used to have friends who were very courageous and enthusiastic when they joined the profession. But now become demotivated and insensitive to patients. I understand them totally.....it is the situation that made them like this… they did not get upgrading chance [professional development opportunities] after serving many years* [IDI code 15]

In addition, minimising contact time with patients was also mentioned as one of the consequences. Some also mentioned that workload was another factor in triggering these reactions in staff. One staff member recalled his personal experience as follows:

*I experienced ‘kesera ga yeteyayaze metaket’ [burnout] once. At that time....there was a community based campaign task and all staff except two HCWs went out for the task. I was with the group that was staying behind in the health centre to provide all the services… here adult, child outpatient service, family planning and antenatal care, delivery you name it… only two individuals were supposed to cover that. No lunch break. We were seeing patients continuously … it was exhausting and stressful. Seeing patient after patient was monotonous.... it makes you passive. … I guess this was when I had the experience of burnout*. [IDI code 04]

Although exposure to stressors for a period of time was seen as inevitable, developing the consequences of prolonged stress exposure was thought to be avoidable by not staying in the same post. Most said that the professionals should make an effort to improve their educational status or change the place of work. Few participants expressed sympathy towards the ‘chronic’ staff. Some tended to blame them for their predicament, on the grounds of personal weakness or laziness, and to feel proud that they themselves were resilient enough not to give way to such challenges and stressors.

*Sometimes there are staff who are not acting like HCWs… he acts carelessly even when he sees the worst cases. I always ask myself why staff are acting like this… become isolated and hostile. I think it is because he wasn’t a good person or he lacked motivation. What I understand is that, he may not achieve his economic desire or he may not be satisfied regarding that….that is why he is so harsh to everyone. I think he is unhappy and he is not motivated in my opinion [because] his income is small… Due to this economic problem he is unhappy and dissatisfied on his job*. [IDI code 11]

*…There are demotivated people who are not interested in the profession; I can understand those who experience burnout due to other economic problems. Apart from this I see it as being lazy. So far I have never experienced it. I hope I will not experience it in the future*. [IDI code 10]

### Theme three: Strategies to handle stressors and their consequences

To handle stress and its impacts, participants reported using or planning to use various strategies. These ranged from enduring the situation to planning to quit the job. Apart from system level changes, participants mentioned inter- and intra-personal factors such as resilience, good peer relationships and social support as important factors for handling stressors or coping. Provision of a good service and avoiding procrastination, especially in relation to paperwork, were some of the strategies used to enhance wellbeing.

*When I make my patients comfortable and treat them with respect we will communicate peacefully … I will get peace of mind and satisfaction. Then I will be well. This is my experience* [FGD one code 06]

Family and having social support were mentioned as important factors to cope with the stressors in the work place. One participant described the advantage of family as follows:

*When I don’t attend some social events or get involved in social activities as I am expected, I have to explain my situation to my family and people around me. Otherwise I will be in trouble, if there is someone who can understand my day-to-day task and what is happening in my workplace that would be great. Because that person will have a positive attitude, he/she will support me. I will not be expected to explain what is going on every day. Having such kind [supportive] of family is useful to work in a place like us*. [FGD two code 01]

Experience sharing with senior staff and getting technical support from other co-workers were other strategies employed. Some participants said that spirituality and prayer helped them to cope with stressful situations. The over-riding coping strategy of HCWs was, however, focused on upgrading their educational status, which would have a direct impact on their career progression and economic security.

Other structural or system level factors that were identified by participants as useful to handle the threats to wellbeing were providing recognition for the HCWs, ensuring adequate numbers of staff, workload reduction, fair career progression, improving the community awareness about their responsibilities as well as their rights, improving public transportation access, and also ensuring adequate remuneration through their salary and benefit packages. Improving communication skills, understanding the culture and norms of the community, explaining what one is doing and discussing plans with patients, preparing social events like coffee ceremonies to share experiences and to refresh themselves were all ways that the HCWs had used to cope with stress and remain mentally healthy.

## Discussion

In order to promote the wellbeing of HCWs in Ethiopia, it is necessary to have a clear understanding of the construct in this sociocultural context, as well as threats to wellbeing and the consequences of those threats. In this exploratory study, wellbeing was largely conceptualised by HCWs as the absence of negative factors rather than as a positive state. An extensive range of threats to wellbeing was identified, spanning the personal and work domains. Core features of burnout were described and portrayed as a consequence of long-term exposure to stressors. Burnout was presented as a problem of others, especially those who have not been able to progress in their careers. Few participants were comfortable to disclose the consequences of long-term exposure to stressors or burnout as a personal experience. Potential targets for intervention were identified. The conceptual framework for this study is illustrated in Fig. 1.

### Wellbeing and threats to wellbeing

Wellbeing is a dimension of health and has been conceptualised in various ways. For some it is related to a state of mind and virtue [46], for others it is the ability to effectively perform some life tasks. Generally speaking, the wellbeing model views the individual in an holistic manner and focuses on the interface between an individual’s physical, social, psychological and spiritual life [47]. Our study participants’ description also shares the reliance of wellbeing on the interface of the individual with their surroundings but it gives greater prominence to threats to wellbeing.

Participants identified a range of threats to their wellbeing and emphasized the interconnectedness of general life and job-related stressors [9, 48]. Exposure to stressors as part of their job was viewed as unavoidable, which is in keeping with previous work from both high income countries [49, 50] and LMICs [12, 16, 51]. Nonetheless, participants distinguished between stressors that were acceptable (related to patent care) and those which were unacceptable (e.g. unable to become economically self-sufficient). Both the physical and the social/relational aspects of the environment were identified as important contributors to wellbeing. The social/relational work environment included workplace relationships with peers, superiors, patients and community members. A previous study found that HCWs friendships were inversely related to job-related stress [52].

New policies to allow patients to express dissatisfaction with the quality of care in a public forum were viewed by most as a potent threat to wellbeing. The experience of being questioned by patients and community members was perceived as being disrespectful to HCWs and undermining of their status. For many of the participants in our study, the traditional status bestowed on HCWs was identified as a motivating factor for pursuing this line of profession despite the stressful environment and limited financial remuneration. However, with the perceived loss of standing in the eyes of the community, many of the HCWs in the study expressed demoralisation.

Participants indicated that the physical demands of the work environment played a significant role in their wellbeing. Availability of medical supplies, tap water, a phone signal and access to public transportation were raised as important factors for doing the job well, protecting their safety and facilitating communication with other HCWs and their family members. Other studies have found that the physical work environment has an influence on HCWs-patients communication and the retention of HCWs, as well as in the promotion of positive outcomes, such as employees’ wellbeing [53], productivity [54] and morale [50].

Another set of threats to wellbeing identified in the literature, particularly in relation to the experience of burnout and job related stress, include job title [55], level of education, job characteristics [14] and socio-economic status (especially wage levels) [6]. Our results also indicated that qualification or belonging to a professional group has an explicit contribution to the experience of job-related stress and burnout. Diploma level nurses seemed to be particularly vulnerable.. Level of income, position, workload, control over the job and lack of career progression opportunities are directly linked to a strong sense of identification with one’s profession. Level of educational progression in the profession also directly impacts the professional’s sense of job security and potential for growth. In contrast, in a quantitative study from a high income country, job characteristics, as well as belonging to different health professional groups, had no significant influence on the experience of job-related emotional strain [7]. Other studies have indicated that long working hours and shift work are associated with impaired HCW wellbeing [56], but were not prominent in the responses of participants in our study.

### “The chronics”

The effects of long-term exposure to stressors, including features of burnout, were viewed negatively and considered as socially unacceptable in this study. Although most of the participants were not familiar with the terms ‘burnout’ or ‘burnout syndrome’, they recognised undesirable consequences for HCWs when they stagnate in one position without educational improvement and career progression. The three dimensional description of burnout was not fully shared by our participants [57]. Although they gave descriptions approximating to all of the dimensions, the personal accomplishment component dominated their discourse. Our study participants stressed that the feeling of personal accomplishment, status and recognition were more important than the experience of emotional exhaustion and depersonalisation. Burnout is associated with various physical and emotional symptoms [58], such as sleep disturbance, headache, hypertension, lower back pain and gastric disturbance among others [59]. However none of the physical symptoms were identified by most of our participants; only one participant described experiencing frequent headaches. The participants extensively discussed the emotional and psychological symptoms such as a sense of hopelessness; irritability and overall dissatisfaction in their professional life.

Due to the stigma or risk of being seen as weak, most of the study participants were not willing to share their experiences and were more comfortable discussing the experiences of other professionals. However, many of the participants admitted to the inevitability of the exposure to the threats of work related stress and burnout, if current contributing factors were not addressed. In a recent study from Northwest Ethiopia HCWs facing dissatisfaction in their income expressed the intention to leave their workplace [51]. A study on PHC workers in Ghana has also indicated there is a strong association between motivation, job dissatisfaction and intention to leave the workplace [16]. A similar finding was reported in a study of rural health workers in Zambia [60]. In our study, most participants reported that the potential for upward career progression and/or change of work place helped them cope with the day-to-day stress of their profession. In the FGD there was one participant who openly spoke about their plan to leave their job and even to change profession.

### Implications for practice

A multi-faceted and contextually adapted approach is required to promote HCW wellbeing. Interventions need to enhance HCW resilience, address the identified threats to wellbeing and support those HCWs who develop burnout or mental health problems. Respondents in this study indicated that strengthening resilience may be achieved through providing opportunities for experience-sharing and shared social activities, such as participation in the Ethiopian coffee ceremony. This result is echoed by a study on the impact of psychosocial support on resilience building [61]. Greater awareness of actions that can be taken to prevent mental illness and promote mental health and wellbeing (e.g. positive coping skills) may also be of benefit [61].

In terms of addressing threats to well-being, our findings indicate that the Ethiopian primary care system requires interventions which target both the health management structure and the work environment. In particular, ways to promote more constructive criticism of health workers during the public evaluation fora may be helpful. The ‘learning health system’ approach, whereby service users are proactively engaged to work with healthcare providers to improve care, has the potential to lead to more productive collaboration and awareness between service providers and service users [62]. Alongside this, necessary environmental interventions include the provision of basic infrastructure such as tap water, electricity and transportation, and ensuring an environment that allows the safe practice of care. Interventions outside of the healthcare system also have the potential to benefit HCW wellbeing, in particular those ensuring that HCWs receive adequate remuneration and opportunities for career development. In a systematic review on interventions to retain HCWs in rural and remote areas of LMICs, to address high turnover and poor quality of the health service [63], financial incentives were important for retention. A programme of continuous professional development is needed for all staff in order to ensure fair career progression, as well as to improve the knowledge and skill of the staff.

Interventions are also needed to address burn-out and other mental health problems in HCWs. Accepting or admitting vulnerability and manifesting emotional consequences of exposure to stressors was seen by almost all respondents as a sign of weakness or defeat, reflecting the stigma associated with mental health problems in Ethiopia [64]. Such attitudes may hinder health providers from sharing their experiences and getting support from their peers or usual social support networks. Therefore, countering stigmatising attitudes towards of burnout and mental health problems is needed. The integration of mental health services in PHC settings in Ethiopia and other LMIC provides an opportunity to raise awareness in HCWs of their own vulnerability to burnout and mental health problems (depression, anxiety, substance use and stress-related conditions) and the availability of treatments. In particular, provision of contextually adapted psychosocial interventions through primary health care facilities may be of benefit to service users and HCWs alike.

### Limitations of the study

We only interviewed two HCWs in a managerial position and may not have adequately captured the perspectives of this group of HCWs. Participants from the same health centre were grouped together for FGDs which meant that some participants might not have felt free to speak about relationships with colleagues and issues that might jeopardise their promotion or standing within the facility. However maximum effort was made to encourage participants to openly share their ideas.

## Conclusions

The findings of this study indicate that threats to health worker wellbeing are commonplace and that burnout is a valid concept in the context of Ethiopian primary healthcare. There is a need for further studies to understand the impact of burnout in relation to staff mental health, job turnover and the quality of care provided, as well as to develop and evaluated contextually appropriate interventions to improve staff wellbeing. Recent initiatives to integrate mental healthcare into primary care may provide an opportunity to promote the wellbeing of healthcare workers and address burnout and emotional problems by creating a better understanding of mental health and opportunities to access care.

## Abbreviations

FGD: Focus Group Discussion
HCW: HealthCare Worker
HEW: Health extension worker
HSTP: Health sector transformation plan
IDI: In-depth interview
LMIC: Low and middle income countries
PHC: Primary health care
PRIME: Programme for improving mental healthcare
SDG: Sustainable development goals
SNNPR: Southern Nations, Nationalities and Peoples Regions
WHO: World Health Organization
